# Artificial Intelligence-Driven Drug Toxicity Prediction: Advances, Challenges, and Future Directions

**DOI:** 10.3390/toxics13070525

**Published:** 2025-06-23

**Authors:** Ruiqiu Zhang, Hairuo Wen, Zhi Lin, Bo Li, Xiaobing Zhou

**Affiliations:** 1 National Institutes for Food and Drug Control, Chinese Academy of Medical-Sciences and Peking Union Medical College, Beijing 100730, China; 2 National Center for Safety Evaluation of Drugs, National Institutes for Food and Drug Control, Beijing 100076, China; 3 State Key Laboratory of Drug Regulatory Science, Beijing 102629, China

**Keywords:** Artificial Intelligence, machine learning, deep learning, toxicity endpoints, transfer learning

## Abstract

Drug toxicity prediction plays a crucial role in the drug research and development process, ensuring clinical drug safety. However, traditional methods are hampered by high cost, low throughput, and uncertainty of cross-species extrapolation, which has become a key bottleneck restricting the efficiency of new drug research and development. The breakthrough development of Artificial Intelligence (AI) technology, especially the application of deep learning and multimodal data fusion strategy, is reshaping the scientific paradigm of drug toxicology assessment. In this review, we focus on the application of AI in the field of drug toxicity prediction and systematically summarize the relevant literature and development status globally in the past years. The application of various toxicity databases in the prediction was elaborated in detail, and the research results and methods for the prediction of different toxicity endpoints were analyzed in depth, including acute toxicity, carcinogenicity, organ-specific toxicity, etc. Furthermore, this paper discusses the application progress of AI technologies (e.g., machine learning and deep learning model) in drug toxicity prediction, analyzes their advantages and challenges, and outlines the future development direction. It aims to provide a comprehensive and in-depth theoretical framework and actionable technical strategies for toxicity prediction in drug development.

## 1. Introduction

Drugs, as chemical substances that can change the physiological or psychological state of organisms, can not only be used for disease prevention, diagnosis, and treatment, improve the quality of life of patients, and even extend the length of human life but also may have different degrees of toxic effects on the body, cause some adverse reactions, cause permanent damage to specific organs, and even cause disability or death of patients. Therefore, determining how to avoid the safety risks of drugs is one of the main challenges faced by drug development, and it is also a crucial and urgent public health problem worldwide [1,2]. In the process of drug research and development, drug toxicity prediction is the key link to ensure drug safety. The toxicity of drugs is not only related to the life and health of patients but also has a decisive impact on the cost, cycle, and marketing approval of drug research and development. Statistical analysis found that among the reasons for the failure of drug research and development, safety factors such as toxicity and side effects accounted for 30% [3,4], making them the main reason for the failure of drug research and development beyond pharmacodynamic factors. Some of the drugs already on the market have also been withdrawn due to major safety problems. The failure of listed drugs will not only lead to huge economic losses but also endanger human health. Therefore, in the early stages of drug research and development, the success rate of innovative drug research and development can be improved by accurately evaluating and predicting the safety of candidate compounds, removing toxic compounds from the candidate compound library, or carrying out targeted structural transformation of toxic compounds.

Traditional methods of drug toxicity prediction are mainly based on in vitro experiments and animal experiments [2,3]. These methods have many limitations, such as long experimental cycles, high costs, and limited prediction accuracy [3]. Although animal experiments can simulate human physiological responses to a certain extent, there are species differences, and the accuracy of extrapolation of experimental results to the human body is limited. Traditional drug toxicity prediction methods can struggle to meet the needs of modern drug research and development. Therefore, AI-enabled drug toxicity prediction technology came into being [5,6], which has brought new opportunities to solve this problem. AI technology, especially machine learning and deep learning algorithms [7], has powerful data processing and pattern recognition capabilities. In drug toxicity prediction, AI can quickly analyze massive data of drug structure, activity, toxicity, etc., and mine the hidden rules and associations so as to establish a high-precision prediction model. Compared with traditional methods, AI technology has higher efficiency and accuracy and can quickly screen potentially toxic drugs in the early stage of drug development, reducing the cost and risk of research and development. At the same time, AI models can also be optimized and updated by constantly learning new data, improving the prediction performance, and providing more reliable support for drug toxicity prediction [1,8].

This review focuses on the application of AI in the field of drug toxicity prediction and systematically combs the relevant literature and development at home and abroad in recent years. The application of various toxicity databases in the prediction was elaborated in detail, and the research results and methods for the prediction of different toxicity endpoints were analyzed in depth, including acute toxicity, carcinogenicity, organ-specific toxicity, etc. At the same time, this paper discusses the application progress of AI technology, such as machine learning and deep learning models, in drug toxicity prediction, analyzes its advantages and challenges, and looks forward to future development directions, aiming to provide a comprehensive and in-depth theoretical reference and technical guidance for toxicity prediction in the process of drug research and development.

## 2. Drug Toxicity Prediction: Databases and Tools

### 2.1. Toxicity Database

#### 2.1.1. Toxicology Resources for Intelligent Computation

The Toxicology Resources for Intelligent Computation (TOXRIC) database is a comprehensive toxicity database, containing a large number of toxicity data of compounds, which are derived from a variety of experiments and literature. Its data cover acute toxicity, chronic toxicity, carcinogenicity, and other types of toxicity, and the species involved include humans, animals, and aquatic organisms [9]. In the prediction of drug toxicity, the TOXRIC database provides rich training data from which researchers can extract the structure of drug molecules and the corresponding toxicity information for the construction and training of machine learning models.

#### 2.1.2. Integrated Chemical Environment (ICE)

The ICE database integrates chemical substance information and toxicity data from multiple data sources [10], including the basic properties of chemical substances (such as molecular formula, structural formula, etc.), toxicological data (such as median lethal dose (LD50), half maximal inhibitory concentration (IC50), etc.) [11], and environmental fate information. The data in this database have high quality and reliability and provide researchers with comprehensive chemical information and toxicity references in drug toxicity prediction research.

#### 2.1.3. Distributed Structure-Searchable Toxicity (DSSTox) Database

The DSSTox database is a large searchable toxicity database that contains a large number of structure, toxicity, and related experimental data on chemical substances [12]. Toxval is part of DSSTox and provides standardized data on toxicity values. These data are widely used in drug toxicity prediction, environmental risk assessment, and other fields. In drug research and development, researchers can use the data in DSSTox and Toxval databases to preliminarily evaluate and screen the toxicity of drug molecules.

#### 2.1.4. DrugBank

The DrugBank database is a comprehensive and freely accessible online database, which provides detailed information on drugs and their targets, including basic data, chemical structure, pharmacological data on drugs, and the sequence, structure, and pathway information of targets [13]. In addition, it also contains clinical information such as clinical trials, adverse reactions, and drug interactions, and provides functions such as external links, data downloads, and Application Programming Interface (API) access.

#### 2.1.5. ChEMBL

The ChEMBL database is a manually curated database of bioactive molecules with drug-like properties. It brings together chemical, bioactive, and genomic data [14]. It aims to translate genomic information into effective new drugs. The database integrates a large amount of data from authoritative journals, patents, and laboratory research reports, providing a wealth of compound structure information, bioactivity data, drug target information, and absorption, distribution, metabolism, excretion, and toxicity (ADMET) data. Researchers can access and query data in many ways, including chemical structure searches, drug name searches, target searches, etc., and use the analysis tools provided by the database for activity clustering, structural similarity searches, and other analyses.

#### 2.1.6. Online Chemical Modeling Environment (OCHEM)

The OCHEM database currently contains 4,064,457 records with 695 attributes, sourced from 20,932 distinct references [15]. Through OCHEM, users can build Quantitative Structure–Activity Relationship (QSAR) models to predict chemical properties or screen chemical libraries according to structural alarms of mutagenicity, skin sensitization, aquatic toxicity, and other endpoints.

#### 2.1.7. PubChem

PubChem is a world-renowned database of chemical substances, which contains massive data on the structure, activity, and toxicity of chemical substances [16]. It integrates information from scientific research literature, experimental reports, and other databases, and has the characteristics of a large amount of data and timely updates. In drug toxicity prediction, the PubChem database is one of the important data sources, from which researchers can obtain a large number of drug molecular data and corresponding toxicity information for model training and validation.

In addition to the seven toxicity databases mentioned above, Table 1 also lists a variety of existing databases related to drug toxicology resources, showing the basic information, data scale, and characteristics of the database. These databases provide all kinds of information about the toxicity of drugs or exogenous substances to cells, organs, or the whole body. Toxicity endpoints include mutagenicity, carcinogenicity, skin sensitization, teratogenicity, endocrine interference, organ-specific toxicity, and other toxicity endpoints. Data types are not only limited to in vivo and in vitro test data but also include predictive data QSAR models.

### 2.2. Biological Experimental Data

#### 2.2.1. In Vitro Cytotoxicity Test Data

The in vitro cytotoxicity test is a common method to evaluate the toxicity of drugs. The toxicity information of drugs at the cellular level can be obtained by detecting the growth inhibition, apoptosis, and other indicators of drugs on cells. These experimental data can be used as the training data of the AI model to construct the cytotoxicity prediction model. Common cytotoxic tests in vitro include the MTT test [17], the CCK-8 test [18], etc.

#### 2.2.2. Animal Experiment Data

Although animal experiments have some limitations in the prediction of drug toxicity, it is still one of the important means to evaluate drug toxicity. Animal experimental data include pharmacokinetic parameters, tissue and organ toxicity, reproductive toxicity, and other information on drugs in animals. These data can provide more comprehensive toxicity information for AI models and help to improve the prediction accuracy of the model. However, when using animal experimental data, attention should be paid to the transformation and application of data to reduce the impact of species differences on the prediction results.

### 2.3. Clinical Data

#### 2.3.1. Food and Drug Administration (FDA) Adverse Event Reporting System

The FDA Adverse Event Reporting System (FAERS) is an adverse drug reaction reporting system established by the FDA [19]. It has collected a large number of adverse drug reaction reports from the drug market. These reports contain information on various toxic reactions of drugs during clinical use, such as drug-induced liver injury, kidney injury, cardiovascular toxicity, etc. By mining FAERS data, we can build a drug toxicity prediction model based on clinical data and provide an important reference for drug safety evaluation.

#### 2.3.2. Electronic Medical Record System

The electronic medical record system (EMR) records the detailed medical information of patients, including drug use records, disease diagnosis, laboratory test results, etc. These data can be used to analyze the toxicity of drugs in practical clinical applications and provide more authentic and comprehensive data support for drug toxicity prediction. However, there are some problems with the quality and integrity of EMR data. It mainly includes the following aspects: (1) inconsistent formats (for example, mixed date/number representations leading to parsing errors), (2) missing key fields (approximately 30% dose omissions, 41% unrecorded concomitant drugs, and 25–60% missing organ toxicity biomarkers), and (3) non-standardized terms. To solve these problems, an integrated preprocessing pipeline is required—standardizing the format through regular expressions, inputting missing values using the k-nearest neighbor algorithm, mapping free text to the standardized ontology through natural language processing (NLP) techniques [20], and filtering out biologically unreliable values—in order to improve the reliability of computational toxicology applications.

**Table 1:** Databases and tools used for chemical toxicity prediction.

Number	Database Name	Database Content	Characteristic	Developer	Website
1	Tox21 [21]	It contains data on more than 10,000 chemicals, as well as nuclear receptors and stress response pathways, generating more than 150 million data points.	High throughput screening data; Multiple toxicological toxicity endpoints; Data visualization and open access	FDA	https://tripod.nih.gov/tox21/challenge/
2	Comp Tox Dashboard [22]	It contains data on more than 1.2 million chemicals and provides information about chemical structure, environmental behavior, and biological activity.	Real-time prediction; Batch Search; Advanced search; Data integration	Environmental Protection Agency (EPA)	https://comptox.epa.gov/dashboard
3	Toxicity Reference Database (ToxRefDB) [23]	It contains in vivo research data of more than 5900 guideline or class guideline studies from more than 1100 chemicals and provides quantitative dose-response data of each dose treatment group, including data of the control group, as well as dose, effect value, and variance information.	High-quality data; All-purpose; Data integration; Distinguishing between missing and negative endpoints	EPA	https://www.epa.gov/chemical-research/downloadable-computational-toxicology-data
4	Toxin and Toxin Target Database (T3DB) [24]	It contains 3678 toxins, described by 41,602 synonyms, including pollutants, pesticides, drugs, and food toxins. These toxins were associated with 2073 corresponding toxin target records, with a total of 42,374 toxin target associations.	Data diversity and accessibility; Potential applications; Data integration	The Chinese University of Hong Kong	http://www.t3db.ca/
5	Therapeutic Target Database (TTD) [25]	TTD contains more than 3500 drug targets and nearly 40,000 drug molecules. The database provides information about target-related diseases and helps researchers understand the potential role of targets in disease treatment.	Reliable data source; Clear target classification; Powerful retrieval function; Rich auxiliary functions	National University of Singapore	http://db.idrblab.net/ttd/
6	Side Effect Resource (SIDER) [26]	It contains information on 1430 drugs and 5868 side effects, covering a variety of treatment areas. The database provides frequency data of each side effect, allowing users to assess the possibility of specific adverse reactions.	Comprehensiveness; Reliable data source; Multilingual support; Structured data	Technical University of Berlin	http://sideeffects.embl.de/
7	The Marker [27]	It contains 218 efficacy biomarkers, 624 safety biomarkers, 104 monitoring biomarkers, 15,893 predictive biomarkers, and 103 alternative endpoints. These data cover a large number of drugs and a wide range of disease categories, not limited to anti-cancer therapy.	Systematic organization; Rich data; User-friendliness; Free access	IDRBLAB	https://idrblab.org/themarker
8	Comparative Toxicogenomics Database (CTD) [28]	CTD contains the interaction information between chemical substances and genes/proteins, as well as the interaction information between chemical substances and phenotypes.	User-friendly interface; The database is searchable, accessible, interoperable, and reusable; Non-redundant data; Comparative analysis is available	North Carolina State University	https://ctdbase.org
9	Gene Expression Omnibus (GEO) [29]	GEO’s significance for ML models stems from its vast repository of gene expression data, encompassing both microarray and RNA-seq datasets. These datasets enable the training of models to predict how chemicals or drugs alter gene expression, thereby providing insights into their potential molecular-level toxic effects.	High data quality; Various forms of data storage; Data visualization; Easy data retrieval; Timely data update	National Center for Biotechnology Information (NCBI)	https://www.ncbi.nlm.nih.gov/geo/
10	Drug Matrix [30]	It contains the comprehensive results of thousands of highly controlled and standardized toxicological experiments involving rat or primary rat hepatocytes, which are systematically treated with therapeutic, industrial, and environmental chemicals, including non-toxic doses and toxic doses	High-quality data; Multiple data types; User-friendliness; Data analysis tools	National Toxicology Program (NTP)	https://norecopa.no/3r-guide/drugmatrix https://ntp.niehs.nih.gov/drugmatrix/index.html
11	Kyoto Encyclopedia of Genes and Genomes (KEGG) [31]	KEGG is an encyclopedia of genes and genomes, which offers structured information on metabolic pathways, molecular interactions, and gene functions.	Highly organized data structure; Multiple data types; Cross-species comparison; Multiple applications	Kanehisa Laboratory	https://www.genome.jp/kegg
12	Universal Protein Database (UniProt) [32]	It is a comprehensive database of protein sequence and function information. It provides detailed annotation of protein function, including protein function, subcellular localization, post-translational modification, protein–protein interaction, and pathway information.	Functional notes; Data consolidation; Sequence similarity search; Proteomic analysis	European Bioinformatics Institute (EBI)	https://www.uniprot.org/
13	Therapeutics Data Commons (TDC) [33]	TDC covers a wide range of learning tasks, including target discovery, activity screening, efficacy, safety, and manufacturing, involving small molecules, antibodies, vaccines, and other biomedical products.	Three-tier structure; Rich datasets; Machine learning tasks; Data processing and evaluation	Harvard University	https://tdcommons.ai/
14	Toxbank [34]	Dedicated database for toxicity data management and modeling repository of “gold” compounds and selected test compounds, as well as reference resources of cells, cell lines, and tissues related to in vitro toxicity studies.	Data management; Interdisciplinary cooperation; Data sharing	EU FP7 project	https://toxbank.net/
15	LiverTox: Clinical and Research Information on Drug-Induced Liver Injury (LIVERTOX) [35]	LiverTox provides the clinical characteristics of drug-induced liver injury, disease severity classification, and causality evaluation scale. The database contains detailed records of more than 1400 drugs, herbs, and dietary supplements. The records of each drug include background information, hepatotoxicity description, case reports, references, etc.	Free use; Timely update; Comprehensiveness; Clinical and research support	National Library of Medicine (NIH)	https://livertox.nih.gov/
16	Gene Expression Nebulas (GEN) [36]	Gen database integrates 323 high-quality transcriptome datasets, covering 50,500 samples of 30 species and 15,540,169 cells, and provides transcriptional maps under six biological scenarios: baseline reference, genetics, phenotype, environment, time, and space.	Data quality control and standardization; Analysis and visualization tools; User-friendliness	National Biological Information Center	https://ngdc.cncb.ac.cn/gen
17	The Cancer Genome Atlas (TCGA) [37]	The TCGA project conducted molecular characterization analysis on more than 20,000 primary cancers and matched normal samples, covering 33 cancer categories. It also contains detailed clinical data, such as patient survival time, treatment response, etc. These data are of great value for the study of clinical characteristics and treatment effects of cancer	Openness and sharing of data; Diversity and comprehensiveness of data; High quality and standardization of data	National Cancer Institute (NCI)	https://www.cancer.gov/ccg/research/genome-sequencing/tcga
18	GeneCards [38]	Genecards provide detailed information about all known and predicted human genes, including genome location, function, expression pattern, genetic variation, clinical relevance, and functional annotation of genes. It also integrates a variety of biological pathway information, providing the role and relationship of genes in different biological pathways	Data consolidation; User-friendliness; Analytical tools; Data access and mining	Weizmann Institute of Science	https://www.genecards.org/

## 3. Application of Artificial Intelligence and Core Algorithm Technology

### 3.1. Machine Learning Algorithm

#### 3.1.1. Traditional Machine Learning Algorithm

**Figure 1:**
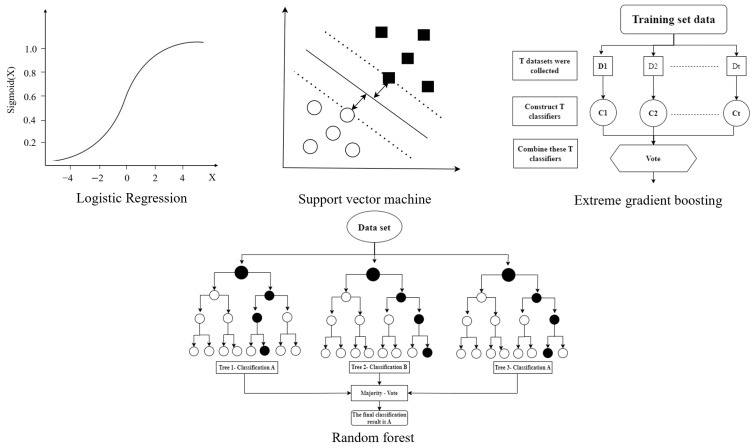
ML algorithm workflow for drug toxicity prediction.

The traditional machine learning (ML) algorithm is a kind of machine learning method used before the rise of deep learning (neural networks). These algorithms usually rely on feature engineering and statistical techniques rather than end-to-end learning methods of deep neural networks. We list three common traditional machine learning algorithms and show their schematic diagrams, as shown in Figure 1.

Logistic regression (LR) is one of the most basic methods in the field of machine learning, and it is also a traditional model often used to interpret clinical trial data in biomedical research [39]. In the prediction of drug toxicity by logistic regression, the characteristics of drugs (molecular descriptors, physical and chemical properties, etc.) are associated with toxicity labels by constructing a linear model to calculate the probability of drug toxicity, which directly shows the weights of chemical descriptors in toxicity predictions [40]. Its advantage is that the model is simple, easy to understand and explain, and can directly display the weight of each feature in toxicity prediction, which has strong interpretability [41]. When predicting the acute toxicity of drugs, the linear relationship between some molecular structure characteristics and acute toxicity can be determined by the LR model. However, LR requires high linear separability of data and has limited ability to model complex nonlinear relationships [42].

Random forest (RF) is an integrated learning method [43]. It is based on multiple independent decision trees and obtains the final output by combining the prediction results of each tree. The algorithm can be used in feature selection, classification tasks, regression analysis, and other fields. At present, the random forest algorithm has been applied to many related works on toxicity prediction, such as the prediction of common side effects of chemotherapy drugs [44], the probability of liver toxicity induced by inhibitors [45], and the risk assessment of drug toxicity [46].

The support vector machine (SVM) is a supervised learning algorithm based on the application of statistical theory [47], which is widely used in classification and regression problems. SVM separates different categories of data by finding an optimal classification hyperplane. The SVM algorithm is used to predict liver injury [48], kidney injury [49], cardiotoxicity [50], immunotoxicity [51], and bone marrow toxicity [52] caused by drugs and their metabolites. The experimental results show that SVM has excellent classification ability in processing small sample toxicity data and has good generalization performance.

Extreme gradient boosting (XGB) is an efficient gradient-lifting decision tree algorithm [53]. In the prediction of drug toxicity, it trains multiple decision trees iteratively to continuously fit the residuals and improve the prediction accuracy of the model. XGB has the advantages of fast computing speed and strong scalability and can handle large-scale datasets. However, the XGB model is relatively complex and struggles to adjust parameters, which requires some experience and skills.

#### 3.1.2. Deep Learning Algorithm

Deep learning (DL) is a special subset of neural networks [54]. It forms a large-scale neural network with multiple hidden layers by deepening the depth of the network and uses the deep neural network (DNN) model to learn. It often supports the processing of thousands of objects and can be competent for more complex work. Raies et al. [55] used three multi-label classification methods (binary association, classifier chain, and unique multi-class) to predict multiple toxicity endpoints of the same compound. The comparative calculation results showed that the classifier chain algorithm had better performance in predicting multiple toxicity effects of drugs. Zhang et al. [56] established a multi-label k-nearest neighbor prediction method based on feature selection combined with multiple algorithms on a dataset containing 1080 drugs and 2260 side effects and integrated the basic classifiers according to the weighted strategy to obtain the final high-precision integration model. Kim et al. [57] analyzed the diversity of pathological findings, collected 21 pathological changes related to 41 drugs, and constructed an integrated model for multi-organ toxicity prediction based on gene expression data. The integrated model is composed of two parts: a k-nearest neighbor classification (KNN) classifier established separately for each pathology and the integrated model of liver or kidney formed by weighted calculation according to the correlation degree between pathology. On the dataset of liver and kidney, the cross-validation of the performance of the integrated model in predicting pathological results showed that the average area under the curve of all single pathological prediction models was 0.68, while the corresponding average of the integrated model was 0.88. In general, the effect of the integrated toxicity prediction model is better than that of the single pathological prediction model.

From this point of view, the development of deep learning algorithms and various interpretable framework algorithms are widely used in drug toxicity and prediction. Next, we will summarize the prediction of specific toxicity endpoints.

### 3.2. Molecular Representation

#### 3.2.1. Molecular Fingerprint

The Morgan fingerprint is a kind of molecular fingerprint based on a circular substructure [58]. It generates substructures by gradually expanding the radius with each atom as the center on the molecular graph and encodes these substructures. Morgan fingerprint can effectively describe the local chemical structure characteristics of molecules and is widely used in similarity searches and model construction in drug toxicity prediction. For example, the potential toxicity of drugs can be preliminarily determined by calculating the Morgan fingerprint similarity between drug molecules and known toxic molecules.

The Molecular Access System (MACCS) fingerprint is a molecular fingerprint based on specific atomic types and substructures. It contains 166 fixed substructure features and has certain chemical significance. In the prediction of drug toxicity, the MACCS fingerprint can be used as a molecular feature representation for model training and prediction. Due to its fixed characteristics, the computational speed is fast, but it may not be able to fully describe the complex structure information of molecules.

The Rdkit fingerprint is a molecular fingerprint implemented in the Rdkit chemical informatics toolkit. It combines a multi-seed structure and topological features and has good versatility and performance. Rdkit fingerprints are widely used in drug toxicity prediction, which can provide rich molecular structure information for the model and help to improve prediction accuracy.

#### 3.2.2. Molecular Descriptor

Molecular descriptors are parameters that quantitatively describe various properties of molecules through mathematical methods, including physical and chemical properties (such as the molecular weight, number of hydrogen bond donors and receptors, lipid water partition coefficient, etc.), topological properties (such as the molecular connectivity index, path length, etc.), and electronic properties (such as charge distribution, frontier orbital energy, etc.). In the prediction of drug toxicity, molecular descriptors can reflect the characteristics of drug molecules from multiple perspectives. As the input of the machine learning model, molecular descriptors can help the model learn the relationship between molecular structure and toxicity.

#### 3.2.3. Molecular Diagram

Molecular graphs represent drug molecules in a graph structure, in which nodes represent atoms and edges represent chemical bonds. Each node and edge can carry attribute information such as atomic type and bond type. Molecular graphs can intuitively display the structure and topology information of drug molecules. In deep learning models, such as the graph neural network (GNN) and gat, molecular graphs can be directly processed and learned by the model as input data. Through the analysis of molecular graphs, the model can capture the interaction and spatial relationship between atoms in molecules so as to predict drug toxicity more accurately. For example, in the GNN-based drug toxicity prediction model, the node features and edge features of the molecular graph are used as the input of the model. After the convolution operation of the multilayer graph, the potential representation of molecules is learned for toxicity prediction. We compared the performance of various molecular representation methods, as shown in Table 2.

## 4. Research Progress of Different Toxicity Prediction

### 4.1. Acute Toxicity Prediction

#### 4.1.1. Definition and Evaluation Index of Acute Toxicity

Acute toxicity describes the harmful effects of a substance that occur shortly after exposure, typically within 24 h [59]. It is commonly evaluated using the LD50, a standard toxicological measure. The LD50 represents the dose of a substance required to kill 50% of a test animal population during a specified observation period. It is a basic measure of the acute toxicity of drugs and plays an important role in drug classification, label management, and preliminary toxicity assessment. According to the regulations of the World Health Organization (WHO), drugs with an LD50 value of less than 5mg/kg are classified as highly toxic drugs, and their safety needs special attention. Common acute toxicity datasets comprise TOXRIC, ICE, EPA’s DSSTox and ToxVal, ChemIDplus, eChemPortal, Japan’s NITE (Chemical Risk Information Platform (CHRIP), PubChem, and researcher-compiled custom datasets.

#### 4.1.2. Relevant Research Results and Methods

The quality and size of acute toxicity datasets are often different, which will affect the robustness of prediction models. Lou et al. integrated the experimental data of 1735 cases of acute toxicity based on rabbits and 1679 cases of acute toxicity based on rats [60] and used machine learning to build a cross-species toxicity prediction model. This study found that in the model based on rat data training, the RF algorithm performed best, and its prediction accuracy was significantly better than that of SVM, XGB, and other control models. In view of the quality differences in the dataset, the research team proposed a two-stage optimization scheme: (1) data screening: eliminating abnormal samples through outlier detection (such as isolation forest) and consistency tests; (2) data enhancement: random transformation of the molecular structure (such as smile enumeration) and feature space expansion (such as molecular descriptor interpolation) technology are adopted to improve data diversity and model generalization ability. This study solves the problem of data heterogeneity in acute toxicity prediction through the strategy of combining data-driven and mechanism interpretation and provides a high-precision and interpretable calculation tool for drug safety assessment. However, due to the need to train multiple basic models, the computational complexity of this model is high, especially when dealing with large-scale datasets. The algorithm requires high data quality and needs data preprocessing and filtering to avoid the influence of noise and outliers. Li et al. developed an integrated learning model [61] based on a super learner to predict the acute toxicity of chemicals to rats. This study integrated the LD50 data of 9843 compounds and constructed 16 meta models using 4 molecular descriptors and a machine learning algorithm. The consistency model achieved R^2^ values of 0.61 (five-fold cross-validation) and 0.64 (test set), with corresponding root mean square errors (RMSEs) of 0.55 and 0.64, respectively. RMSE is a standard metric used to quantify the accuracy of predictive models. It measures the average magnitude of prediction errors between observed values and model-predicted values [62]. These results significantly outperform those of any single benchmark model. By integrating multiple base models, the consistency model enhances both the generalization capability and prediction accuracy. At the same time, it can flexibly select and adjust the basic model according to different datasets and problems. Feitosa et al. developed a machine learning tool called cyto-safe for the early identification of cytotoxic compounds [63]. In this study, the near-miss V3 undersampling method was used to balance the number of toxic and non-toxic samples, the ecfp4 molecular fingerprint was used to represent the structure of the compound, and the light Gradient-Boosting Machine (GBM) algorithm was used to build the model. Under a 1:5 undersampling ratio, the model performed best, with a sensitivity of 83%, and achieved high accuracy in the identification of cytotoxic compounds. It also constructs an interpretable prediction framework, which visually shows the relationship between molecular structure and toxicity prediction through a thermodynamic diagram. However, the dataset was limited to 3T3 and HEK 293 cell lines and did not cover other types of cells.

ML models can extract features from a variety of data sources and transform them into numerical information that can be used for toxicity prediction. Research shows that the RF model performs well in acute toxicity prediction and can effectively deal with complex toxicity data. DL, as a sub-field of machine learning, has surpassed traditional machine learning methods in the field of toxicity prediction because it can automatically construct complex features. For example, graph convolutional networks (GCNs) show stronger applicability in processing complex protein interaction network data.

### 4.2. Organ-Specific Toxicity Prediction

**Figure 2:**
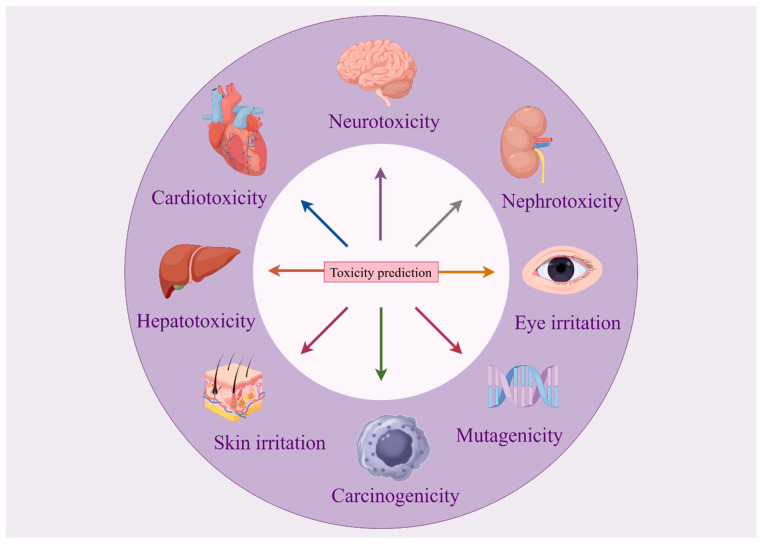
Schematic diagram of drug organ toxicity.

Organ-specific toxicity is critical, as drugs often target particular tissues or organs, causing adverse reactions due to local accumulation or organ-specific sensitivity. Drug-induced organ toxicity encompasses hepatotoxicity, cardiotoxicity, nephrotoxicity, respiratory toxicity, and neurotoxicity, as shown in Figure 2.

#### 4.2.1. Hepatotoxicity

The liver is the main organ of drug metabolism, and hepatotoxicity is one of the common adverse reactions of drugs. The mechanisms of hepatotoxicity include drug-induced liver injury (DILI), interference with liver metabolic function, and immune-mediated liver injury. Hepatotoxicity is one of the most common and serious drug toxicity. The accurate prediction of drug hepatotoxicity is very important to ensure the safety of patients. AI technology has been widely used in the prediction of liver toxicity. By analyzing the structural characteristics of drug molecules, metabolic pathways, and the interaction with liver-related targets, the prediction model is constructed. Chen et al. developed ResNet18DNN, a deep neural network utilizing composite structural images to enhance hepatotoxicity prediction performance [64]. Separately, Füzi et al. proposed a systems biology approach integrating biological pathway-related descriptors, demonstrating superior predictive capability compared to traditional machine learning models such as Gradient-Boosting Trees (GBTs) and random forests (RFs) [65]. Xu et al. [66] used DL to establish a DILI prediction model with chemical structure data. In this study, a recursive neural network was used to build the model. The training was conducted on 475 drugs, and the external validation set of about 200 drugs was predicted. The accuracy, sensitivity, and specificity were 86.9%, 82.5%, and 92.9%, respectively. The performance of this model is better than the DILI prediction model previously reported. Lee et al. [67] employed machine learning models—including random forest (RF), logistic regression (LR), and Neural Networks (NNs)—to predict DILI, identify key molecular descriptors, and enhance model interpretability through feature importance ranking and LR coefficients.

In summary, drug hepatotoxicity prediction is primarily approached as a classification task, though data quality challenges complicate the identification of truly DILI-free drugs. Drug dosage, formulation, and patient baseline conditions can all influence prediction outcomes. Future research will focus on leveraging ML and DL for hepatotoxicity prediction through multi-source data integration, enhanced model interpretability, optimized deep learning architectures, multiclass and hierarchical prediction, personalized precision toxicology, and clinical/regulatory applications.

#### 4.2.2. Nephrotoxicity

The kidney plays a key role in drug excretion, and nephrotoxicity is an important issue in drug research and development [68,69]. The mechanisms of drug-induced nephrotoxicity include the damage to renal tubular epithelial cells, the influence on glomerular filtration function, and the initiation of renal interstitial inflammation. In medical practice, the use of diuretics [70], antibiotics [71,72], and other drugs [73], as well as various imaging examinations, chemotherapy, radiotherapy, and interventional therapy, may lead to nephrotoxicity, which means that nephrotoxicity is difficult to capture. Therefore, we urgently need to build a model that can effectively predict drug-induced nephrotoxicity in advance, so as to achieve early intervention and nursing for patients. In the research on nephrotoxicity prediction, machine learning methods [74,75,76], such as random forest and logistic regression, combined with molecular descriptors and physical and chemical properties, are used in predictions. In recent years, deep learning models have also been gradually applied to the prediction of nephrotoxicity [77,78], such as the model based on convolutional neural networks (CNNs) [79], which extracts features for the prediction of nephrotoxicity via convolution operations on the two-dimensional structure image of drug molecules. At the same time, researchers have also tried to use metabonomic data [80] to analyze the relationship between small-molecule metabolites produced by drug metabolism in vivo and nephrotoxicity, providing new ideas for the prediction of nephrotoxicity. Banerjee et al. employed the Classification Read-Across Structure–Activity Relationship (c-RASAR) approach to develop machine learning models using a recently curated dataset of orally active drugs’ nephrotoxicity potential [76]. Their analysis revealed a strong overall performance for the c-RASAR models. The top-performing model, an LDA-based c-RASAR model utilizing topological descriptors, achieved Matthews Correlation Coefficient (MCC) values of 0.229 (training set) and 0.431 (test set). This model successfully predicted nephrotoxicity in a true external dataset derived from known nephrotoxic compounds in DrugBank DB, demonstrating good predictivity. Rao et al. collected a dataset of 360 FDA-classified compounds (231 non-nephrotoxic and 129 nephrotoxic) and predicted 6064 non-targeted interactions, of which 59% were validated in vitro. At the same time, they also calculated 55 physicochemical properties of these compounds. Multiple algorithms such as SVM, RF, and NN were used to establish ML models. They then combined these models into an integrated model to enhance performance [74]. The model they developed provides a promising early screening tool for identifying compounds with lower risk, thereby promoting safer drug development.

In summary, using clinical data and electronic health records, ML models can accurately predict acute kidney injury by learning and analyzing data patterns [81,82,83]. With the continuous development of new DL models, the integration of multimodal data [84] (such as images and physiological parameters) will be able to improve the performance of nephrotoxicity prediction.

#### 4.2.3. Cardiotoxicity

Drug cardiotoxicity is one of the main reasons for drug development failure and delisting after-market launch. According to FDA statistics, about 30% of drugs are terminated or restricted in clinical trials or post-market stages due to cardiac safety issues, resulting in economic losses exceeding $10 billion annually. Cardiac toxicity is mainly manifested as QT interval prolongation (caused by hERG channel inhibition) [85], cardiomyopathy, arrhythmia, etc. Its prediction and evaluation are the core challenges of drug development. AI-assisted drug toxicity discovery is an effective solution to reduce costs and facilitate the development of lead drug candidates. The most used cardiotoxicity datasets are Tox21, ChEMBL, ICE, PubChem, and hERGCentral. Yang et al. developed a large Language Model as Tools for Molecular Toxicity Prediction to predict cardiotoxicity [86]. They used GPT-4 combined with molecular docking technology to study the cardiotoxicity of three specific targets and examined Chinese medicinal materials listed as food and drugs. This study emphasizes the potential of ChatGPT in predicting molecular properties and its importance in medicinal chemistry, demonstrating that it promotes a new research paradigm: with datasets, high-precision learning models can be generated without computational knowledge or coding skills, making them easy to access and use. The human ether-a-go-go-related gene (hERG) potassium channel is pivotal in drug discovery due to its susceptibility to blockage by drug candidate molecules, which can cause severe cardiotoxic effects. Consequently, identifying and excluding potential hERG channel blockers at the earliest stages of drug development is crucial. To address the need for more precise, quantitative predictions, they developed hERGBoost [87], a cutting-edge machine learning model employing a gradient-boosting algorithm. This model demonstrates superior accuracy in predicting the IC50 of drug candidates. Numerous studies are continuously innovating technology to more accurately predict cardiac toxicity [88,89,90,91]. AI is expected to improve the accuracy of preclinical prediction of cardiac toxicity to over 90%, significantly reducing drug development risks and patient safety threats.

Above all, cardiovascular toxicity is a well-known complication of thoracic radiotherapy (RT), which leads to an increase in incidence rate and mortality. However, existing techniques for predicting cardiovascular toxicity have limitations. Predictive biomarkers [92] for cardiovascular toxicity may help maximize patient prognosis.

#### 4.2.4. Neurotoxicity

Neurotoxicity involves the effects of drugs on the nervous system, which may lead to adverse consequences such as nerve damage and cognitive impairment. The core mechanisms of drug neurotoxicity (such as Parkinson’s-like injury and Alzheimer’s disease-related pathology) involve blood–brain barrier penetration, neurotransmitter interference (such as dopamine/glutamate imbalance), mitochondrial dysfunction, and microglial activation. Zhao et al. utilized real-world human clinical data to extract drug neurotoxicity information, developing 35 distinct classifiers by combining five machine learning methods with seven molecular fingerprinting techniques [93]. Among these, the MACCS-SVM model demonstrated optimal performance. Their analysis identified 18 structural alerts (SA) linked to neurotoxicity, offering interpretable insights. By leveraging both the three-dimensional structural information of drug molecules and their biological activity data on nerve cells, this approach enables the effective identification of compounds with potential neurotoxicity risks, thereby supporting the development and safety assessment of neuro pharmaceuticals. There are fewer neurotoxicity datasets, and commonly used datasets for neurotoxicity prediction are SIDER and PubChem. Chemical-induced neurotoxicity is a key aspect of chemical safety assessment. Traditional and expensive experimental methods require the development of high-throughput virtual screening. However, the small neurotoxic datasets limit the application of advanced deep learning techniques. Pang et al. developed a hybrid deep learning architecture called NeuTox 2.0 [94], which combines transfer learning based on self-supervised learning, graph neural networks, and molecular fingerprints/descriptors. Compared to traditional machine learning, NeuTox 2.0 has excellent noise resistance and high prediction accuracy. NeuTox 2.0 can be used as an effective tool for early neurotoxicity screening of environmental chemicals. HE et al. [95] combined various machine learning algorithms with molecular representations based on Particle Swarm Optimization-weighted scores to construct an ensemble prediction model called NeuTox. For the test set, NeuTox demonstrated excellent performance with an accuracy of 0.9064, outperforming the best-performing individual model. Identifying the structural features of chemical neurotoxicity facilitates the early design of non-toxic chemicals.

In conclusion, traditional compound toxicity detection techniques generally require the use of biochemical experiments, cell experiments, and even animal models, which not only consume a lot of time but also have high costs. The use of computational models for the toxicity prediction of organic compounds requires less input but yields enormous output. In particular, computational models based on the physical, chemical, and structural properties of compounds can even predict them before synthesis, greatly improving efficiency and making them increasingly popular.

#### 4.2.5. Other Toxicities

Mutagenicity refers to the ability of chemical substances to induce gene mutations, which may lead to hereditary Deoxyribonucleic acid (DNA) damage and increase the risk of cancer. Standard assessment methods include the Ames test [96], which measures reverse mutations in strains (such as Salmonella typhimurium). The key databases for predicting mutability include OCHEM, which is a prediction based on structural alerts on the basis of QSAR. Rane et al. used the QSAR method to predict the mutagenicity of gliazoleozinc dimer impurities and simultaneously conducted in vitro evaluations through Ames and micronucleus tests [97]. The mutagenicity of dimer impurities in the in vitro method and QSAR analysis was consistent with the computer prediction. Moreover, Carcinogenicity refers to the possibility of chemical substances inducing tumors. Trihalomethanes (THMs) in drinking water are regulated for carcinogenic health risks. However, frequent water quality monitoring imposes significant resource burdens. Li et al. proposed a framework [98] that combines interpretable machine learning with virtual data augmentation to predict the occurrence of THM and related cancer risks. These findings support data-driven soft sensing for water quality and health risk management in data-limited areas. Moreover, skin Corrosion/Irritation (Corr./Irrit.) has long been a health hazard in the Globally Harmonized System (GHS). Huang et al. have established several computer models to predict skin allergies as an alternative to increasingly limited animal experiments [99]. We believe that the establishment of databases and computer models can facilitate the assessment of chemical safety and related research. Serious eye damage and eye irritation have been authenticated to be significant human health issues in various fields such as ophthalmic pharmaceuticals. Due to the shortcomings of traditional animal testing methods, in silico methods have advanced to study eye toxicity. Di et al. developed models for predicting severe eye damage and eye irritation potential of compounds using 2299 and 5214 compounds, respectively. The test accuracy of the best serious eye damage model and eye irritation model reached 0.972 and 0.959, respectively [100]. In total, the prediction models and structural alerts contributed to providing hazard identification and assessing chemical safety.

## 5. Challenges and Prospects

While AI-driven toxicity prediction offers transformative potential, critical limitations in data quality, model design, and translational validation hinder its reliability in real-world drug development. These challenges demand urgent interdisciplinary collaboration.

### 5.1. Expanding Sample Sizes

Insufficient sample size constitutes a primary challenge, compromising model training and adversely impacting prediction accuracy and generalization capability. As noted, toxicity endpoint labels (e.g., clinical toxicity, nephrotoxicity) remain scarce. However, advances in sequencing technology are rapidly generating multi-omics data and drug perturbation-based toxicity datasets [101]. Machine learning techniques—including data augmentation, pretrained models, active learning, and meta-learning—can mitigate data limitations [102]. Furthermore, data sharing and integration initiatives substantially expand sample sizes. Platforms like CTD and TTD aggregate multi-source data, while public participation and open-access policies accelerate dataset growth. Collectively, these strategies enhance the performance and reliability of ML in toxicity prediction.

### 5.2. Improving Data Quality and Integrating Diverse Sources

The scarcity of high-quality data and the challenges of integrating multi-source datasets have hindered the broader application of machine learning. To address this, data from diverse sources must be standardized using relatively uniform approaches to ensure interoperability. Implementing comprehensive, rigorous data-cleaning and cross-validation processes is essential to minimize noise and errors. Integrating heterogeneous data sources for toxicity prediction faces significant hurdles, including data quality issues, heterogeneity, and sparsity. Ensemble learning methods, along with techniques such as federated learning (FL) and multimodal ML, facilitate the integration of toxicological, chemical, and multi-omics data, helping to mitigate data scarcity [103]. Synthetic data generation technology creates artificial data that simulate the features of unknown categories. Models trained on this synthetic data, which share statistical attributes with real-world data, show improved performance on actual datasets. This technology provides an effective solution to data scarcity and enhances the model’s capability to identify unseen categories. Personalized toxicity prediction, leveraging patient-specific data or synthetic data generation, further addresses data shortages. Collectively, these methods enable the effective combination and enhancement of multi-source datasets, creating unified, high-quality data resources.

### 5.3. Enhancing ML Models for Diverse Toxicity Detection

Predicting multiple toxicities (such as acute toxicity, carcinogenicity, and organ-specific toxicity) is challenging due to their respective unique biological mechanisms, which require the use of specialized models. The prediction of different toxicity endpoints usually requires different feature sets. For example, chemical toxicity might be more suitable to be characterized by molecular descriptors [104]. However, biological toxicity depends more on the gene expression profile [105]. Take liver toxicity as an example. Its prediction requires the integration of liver-specific multi-omics data such as metabolic enzyme activities, bile acid circulation, and oxidative stress and the combination of dynamic response models. Therefore, to further improve the prediction performance, it is necessary to construct appropriate feature representations and dedicated models for various toxicities. DL models offer new opportunities to address the challenges of various toxicity predictions. Compared with traditional methods, DL technology can capture complex nonlinear relationships in data more effectively and has excellent scalability, capable of handling large datasets—which is particularly important for tasks involving high-dimensional chemical and biological data. The key to developing a DL workflow that integrates patients’ genetic information and clinical data to achieve personalized prediction and optimize treatment outcomes lies in promoting interdisciplinary collaboration among toxicologists, data scientists, biologists, and clinicians. More benchmark databases are urgently needed to construct and validate toxicity prediction models. However, existing databases often hinder repeatability and model comparison due to the lack of standardization. Shared and well-planned benchmarks (such as TOXRIC, TDC, and MoleculeNet) can enhance the consistency of evaluations. We encourage researchers to enhance repeatability through open-source code, hyperparameters, and data preprocessing steps on platforms such as GitHub and Zenodo. Ultimately, formulating standardized benchmarks and reporting guidelines is crucial for ensuring the reliability of results and accelerating progress in the field.

### 5.4. Enhance the Interpretability of ML in the Prediction of Drug Toxicity

In drug toxicity prediction, a major challenge for ML models is their lack of interpretability. The core objective of explainability is to present a transparent and traceable prediction process, enabling researchers to understand model decision-making. This enhances the credibility, usability, and acceptability of models in clinical decision-making and regulatory approval, which is crucial for related work.

While advanced interpretability methods like Shapley additive explanations (SHAPs) [106] can handle multiple data types, they often require customization when applied to complex biological data (e.g., time series, multimodal, and high-dimensional sparse data). Furthermore, the absence of widely accepted evaluation criteria makes it difficult to quantify or assess these methods effectively. Current evaluations are largely limited to simple qualitative analyses or narrow-scoped quantitative metrics, falling short of practical application demands [107]. The inherent diversity and complexity of biomedical data (genomic, omics, and clinical) further compound these challenges.

As artificial intelligence overcomes these hurdles and aligns with regulatory frameworks, its potential in regulatory toxicology is being realized. For ML models to meet regulatory requirements, they must ensure transparency and repeatability, gaining approval through standardized processes. AI promotes the development of reliable in vitro and computational models, potentially reducing reliance on animal experimentation. These advancements have cemented machine learning’s potential as a cornerstone of modern regulatory toxicology practice [108]. Integrating multi-omics and chemical data improves toxicity prediction accuracy and toxicological mechanism understanding. Automated, interpretable models can assist regulatory agencies in prioritizing chemicals based on quantitative predictions and guiding subsequent testing. These advancements have cemented machine learning’s role as a cornerstone of modern regulatory toxicology practice [109].

## 6. Conclusions

In this review, we emphasize the increasingly critical role of AI in addressing challenges associated with drug toxicity prediction. By classifying diverse drug-induced toxicities and analyzing their characteristics alongside predictive models, we demonstrate how ML bridges the gap between predictive accuracy and mechanistic understanding. Furthermore, we comprehensively summarize key databases and tools relevant to drug toxicity, providing researchers with valuable resources to explore toxicity across various domains and facilitating data-driven insights for informed decision-making. These advances highlight ML’s potential to overcome limitations inherent in traditional toxicity assessment methods—such as high costs, ethical concerns, and cross-species variability. Despite this progress, significant challenges persist, including data scarcity, model interpretability, and multi-source data integration. Addressing these issues necessitates the continual development of robust, interpretable ML models underpinned by standardized evaluation metrics.

## Figures and Tables

**Table 2:** Molecular representation methods for drug toxicity prediction.

Category	Method	Core Principle	Key Features
Molecular Fingerprint	Morgan Fingerprint	Circular substructure expansion around each atom	- Radius-based atom environments - Encodes local chemical patterns
	MACCS Fingerprint	166 predefined substructure keys	- Fixed binary features - Chemically meaningful fragments (e.g., aromatic rings)
	RDKit Fingerprint	Hybrid of path-based and topological patterns	- Customizable bit length - Combines structural/topological diversity
Molecular Descriptor	Physicochemical	Mathematical quantification of properties	- MW, LogP, H-bond donors/acceptors - Polar surface area
	Topological	Graph-theory indices	- Connectivity indices - Path lengths, branching degrees
	Electronic	Quantum chemical properties	- Partial charges - Frontier orbital energies
Molecular Graph	GNN	Atoms = Nodes, Bonds = Edges	- Node attributes: Atom type, charge - Edge attributes: Bond order, stereo

## Data Availability

No new data were created or analyzed in this study. Data sharing is not applicable to this article.

## List of Abbreviations

The list of abbreviations is shown as follows.

**Full Term**	**Abbreviation**
Absorption, Distribution, Metabolism, Excretion and Toxicity	ADMET
Application Programming Interface	API
Artificial Intelligence	AI
Classification Read-Across Structure-Activity Relationship	RASAR
Comparative Toxicogenomics Database	CTD
Convolutional Neural Network	CNN
Deep Learning	DL
Deep Neural Network	DNN
Deoxyribonucleic Acid	DNA
Distributed Structure-Searchable Toxicity Database	DSSTox
Drug-Induced Liver Injury	DILI
Electronic Medical Record	EMR
Environmental Protection Agency	EPA
European Bioinformatics Institute	EBI
Extreme Gradient Boosting	XGB
FDA Adverse Event Reporting System	FAERS
Federated Learning	FL
Food and Drug Administration	FDA
Gene Expression Nebulas	GEN
Gene Expression Omnibus	GEO
Globally Harmonized System	GHS
Gradient Boosting Machines	GBM
Gradient Boosting Trees	GBT
Graph Convolutional Networks	GCN
Graph Neural Network	GNN
Half Maximal Inhibitory Concentration	IC50
Integrated Chemical Environment	ICE
K-Nearest Neighbor	KNN
Kyoto Encyclopedia of Genes and Genomes	KEGG
LiverTox: Clinical and Research Information on Drug-Induced Liver Injury	LIVERTOX
Logistic Regression	LR
Machine Learning	ML
Matthews Correlation Coefficient	MCC
Median Lethal Dose	LD50
Molecular Access System	MACCS
National Cancer Institute	NCI
National Institute of Technology and Evaluation	NITE
National Library of Medicine	NIH
National Toxicology Program	NTP
Natural Language Processing	NLP
Neural Network	NN
Online Chemical Modeling Environment	OCHEM
Quantitative Structure-Activity Relationship	QSAR
Radio Therapy	RT
Random Forest	RF
Root Mean Square Errors	RMSEs
SHapley Additive exPlanations	SHAP
Side Effect Resource	SIDER
Structural Alarms	SA
Support Vector Machine	SVM
The Cancer Genome Atlas	TCGA
Therapeutic Target Database	TTD
Therapeutics Data Commons	TDC
Toxicity Reference Database	ToxRefDB
Toxicology Resources for Intelligent Computation	TOXRIC
Toxin and Toxin Target Database	T3DB
Trihalomethanes	THMs
Universal Protein Database	UniProt
World Health Organization	WHO
